# Exploring genetic diversity and population structure in *Cinnamomum cassia* (L.) J.Presl germplasm in China through phenotypic, chemical component, and molecular marker analyses

**DOI:** 10.3389/fpls.2024.1374648

**Published:** 2024-07-03

**Authors:** Panpan Han, Jinfang Chen, Zeyu Chen, Xiaoying Che, Ziqiu Peng, Ping Ding

**Affiliations:** College of Traditional Chinese Medicine, Guangzhou University of Chinese Medicine, Guangzhou, China

**Keywords:** *Cinnamomum cassia* (L.) J.Presl, cinnamon, volatile oil, single-nucleotide polymorphism, genetic diversity, population structure, germplasm resources of traditional Chinese medicine

## Abstract

*Cinnamomum cassia* (L.) J.Presl, a tropical aromatic evergreen tree belonging to the Lauraceae family, is commonly used in traditional Chinese medicine. It is also a traditional spice used worldwide. However, little is currently known about the extent of the genetic variability and population structure of *C. cassia*. In this study, 71 individuals were collected from seven populations across two geographical provinces in China. Nine morphological features, three chemical components, and single nucleotide polymorphism (SNP) markers were used in an integrated study of *C. cassia* germplasm variations. Remarkable genetic variation exists in both phenotypic and chemical compositions, and certain traits, such as leaf length, leaf width, volatile oil content, and geographic distribution, are correlated with each other. One-year-old *C. cassia* seedling leaf length, leaf width, elevation, and volatile oil content were found to be the main contributors to diversity, according to principal component analysis (PCA). Three major groupings were identified by cluster analysis based on the phenotypic and volatile oil data. This was in line with the findings of related research using 1,387,213 SNP markers; crucially, they all demonstrated a substantial link with geographic origin. However, there was little similarity between the results of the two clusters. Analysis of molecular variance (AMOVA) revealed that the genetic diversity of *C. Cassia* populations was low, primarily among individuals within populations, accounting for 95.87% of the total. Shannon’s information index (I) varied from 0.418 to 0.513, with a mean of 0.478 (Na=1.860, Ne =1.584, Ho =0.481, He =0.325, and PPB =86.04%). Genetic differentiation across populations was not significant because natural adaptation or extensive exchange of seeds among farmers between environments, thus maintaining the relationship. Following a population structure analysis using the ADMIXTURE software, 71 accessions were found to be clustered into three groups, with 38% of them being of the pure type, a finding that was further supported by PCA. Future breeding strategies and our understanding of the evolutionary relationships within the *C. cassia* population would benefit greatly from a thorough investigation of phenotypic, chemical, and molecular markers.

## 1 Introduction

*Cinnamomum cassia* (L.) J.Presl, a tropical aromatic evergreen tree belonging to the Lauraceae family, is commonly used in traditional Chinese medicine. It is also a traditional spice used worldwide (National Pharmacopoeia Committee, 2020). *Cinnamomum cassia* Presl is derived from the bark of the tree trunk and is used extensively worldwide owing to its brilliant flavor and smell. It has great economic value and is useful not only as a daily condiment but also as a raw ingredient for pharmaceuticals. More than 160 compounds have been isolated and identified from *C. cassia* as a result of the numerous investigations that have been conducted on the pharmacology and phytochemicals of this plant. The primary chemical components of its volatile oils possess anti-inflammatory, antibacterial, and anticancer properties (Shen et al., 2021). Numerous studies have demonstrated the broad spectrum of pharmacological effects of *C. cassia*, including effects against tumors, reduction of inflammation and pain, diabetes mellitus and obesity, prevention of bacteria and viruses, protection of the cardiovascular system, cytoprotection, neuroprotection, and immunoregulatory responses (Kwon et al., 2006; Hong et al., 2012). *C. cassia* is found in China, India, Vietnam, Indonesia, and other countries.As a major ingredient in traditional Chinese medicine, *C. cassia* is typically used throughout Asia. More than 500 formulations containing *C. cassia* are used to treat various illnesses, including inflammatory diseases, chronic gastrointestinal diseases, gynecological disorders, and cardiovascular diseases. *C. cassia* has been listed in the People’s Republic of China Pharmacopoeia (CH.P) since 1963. In China, Guangdong and Guangxi have the largest cultivated areas of *C. cassia*, and the *C. cassia* planting area of Guangdong Province accounts for more than 30% of the country’s planting area. Guangdong’s *C. cassia* is mainly distributed in the Zhaoqing and Yunfu cities, and the cultivated *C. cassia* in these two cities is referred to as “Xijianggui.” Guangxi’s *C. cassia* planting area accounts for more than 50% of the country’s planting area, and Fangchenggang, Dongxing, Yuli, Guiping, and Beiliu cities are the main producing areas of *C. cassia*. Among them, Fangchenggang and Dongxing predominantly cultivate a variety known as “Fangchenggui”, while Yulin and Guiping mainly cultivate the “Xijianggui” variety (Yang et al., 2020; Li et al., 2023).

A key component of breeding operations is gathering and studying germplasm resources. Although they are currently in danger of becoming extinct, the wild resources of *C. cassia* are vital to scientific research and applications because they have mostly been subjected to natural selection and have been little affected by artificial selection. Most *C. cassia* has been sexually propagated, mostly through seeds, and has been grown artificially for many years. Germplasm diversity is the foundation for preventing genetic erosion and gives plant breeders the chance to create new varieties and enhance them with superior features. Previous studies have demonstrated notable variations in the chemical and physical characteristics of various *C. cassia* germplasm resources. For example, the leaves of *C. cassia* from Guangdong Province are larger and contain higher amounts of volatile oil, whereas the leaves from Guangxi are noticeably smaller and contain less volatile oil, with the exception of a few *C. cassia* cultivation areas (Tung et al., 2010; Liang et al., 2016; Li et al., 2021; Narayanankutty et al., 2021; Chen et al., 2022). It is important to analyze genetic diversity and population structure to design breeding methods and research the genetic links of *C. cassia* plants. Molecular markers have become powerful tools for genetic research on *C. cassia* populations, including simple sequence repeats (SSR), inter-SSR (ISSR), internal transcribed spacer (ITS), and *psbA-trnH* (Liang et al., 2016; Wang, 2022). These studies indicate that *C. cassia* populations are highly genetically diverse. However, the populations of *C. cassia* used in earlier research had either limited sample sizes or were relatively sparsely distributed geographically, with Guangdong and Guangxi being the two locations that produced *C. cassia*. It is possible that the current Chinese *C. cassia* cultivars’ limited genetic base results from the utilization of a small number of parental genotypes. Consequently, to increase the genetic diversity of improved varieties, it is imperative to look for additional breeding materials in China. To the best of our knowledge, no prior research has utilized single nucleotide polymorphism (SNP) markers to investigate the genetic diversity and population structure of *C. cassia* germplasm. SNP markers are representative of the third generation of molecular marker technology, which generally refers to DNA sequence polymorphisms caused by the mutation of a single base at the genomic level (Belaj et al., 2018). Currently, SNP molecular markers are considered the most promising molecular markers. They have the advantages of abundance, wide distribution, low mutation frequency, and high genetic stability, among others. SNP markers are used to differentiate and identify extensive plant and animal germplasm resources (Maroso et al., 2019; Sun et al., 2020; Perez et al., 2021). Moreover, they are more directly comparable between different genotypes; therefore, they are more suitable for the genetic analysis of complex and diverse traits. They also contribute to the identification of genes that cause population differences (Ren et al., 2013; Maroso et al., 2019; Nantawan et al., 2019). To successfully utilize invaluable germplasm resources in future breeding efforts, both within China and worldwide, it is mandatory to gain insight into the genomic differentiation and variation of *C. cassia* genotypes.

In this study, we collected 71 germplasm resources of *C. Cassia* across Guangdong and Guangxi. Morphological, biochemical, and molecular markers were used to study the germplasm variation of *C. cassia* comprehensively. The objective of this study was to identify and use diverse gene and genotype resources, investigate the genetic differentiation and population structure of *C. cassia*, clarify the phylogenetic relationships of *C. cassia* from different locations, search for superior *C. cassia* accessions, open up new breeding opportunities, and safeguard germplasm resources.

## 2 Materials and methods

### 2.1 Plant materials

**Figure 1:**
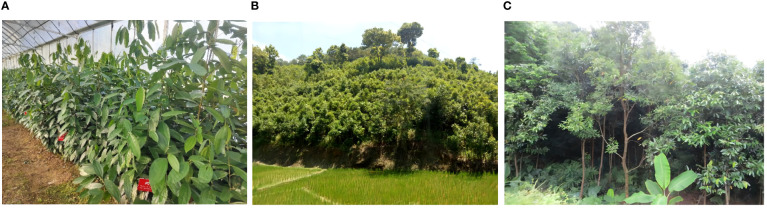
The phenotypic characters of *C. cassia* in different populations. **(A)** The germplasm resource nursery in Deqing county, Guangdong province; **(B)** The *C. cassia* form Guangdong province; **(C)** The *C. cassia* form Guangxi Zhuang Autonomous Region.

Seventy-one *C. cassia* accessions (RG01–RG71) were collected from three populations in Guangdong (Deqing County, Gaoyao County, and Luoding City) and four populations in Guangxi (Guiping, Pingnan, Yulin, and Fangchenggang) (**Supplementary Table S1**). Fifty-four accessions were from Guangdong Province, whereas the rest originated in Guangxi. Simultaneously, the seeds of each sample were planted in a germplasm resource nursery located in Deqing County, Guangdong Province (**Figure 1A**). Morphological data of the one-year-old *C. cassia* seedlings in the resource nursery were recorded later in this study to remove the influence of the ecological environment on the samples. The sample named RG38 was lost, and subsequent analyses, except for the biochemical analysis, did not include RG38. Samples were identified by Professor Ping Ding (Guangzhou University of Chinese Medicine, Guangdong, China).

In a previous study, our research group found that the cinnamaldehyde content in different samples of the same batch of cinnamon medicinal materials was considerably different, and that the volatile oil content between different thicknesses from the same tree was irregular. Therefore, we collected another12 cassia bark samples from the Hetai, Gaoliang, and Tanlin Towns in Guangdong Province to determine the content of the volatile oils cinnamaldehyde, cinnamyl alcohol, cinnamic acid, and 2-methoxycinnamaldehyde (**Supplementary Table S2**). During sampling, cinnamon trees with a growth age of 8 years andheight of approximately 5 m were selected, and the fixed sampling time was October. The tree bark was cut and peeled from 10 cm above the ground, and the bark of each tree trunk was evenly divided into six parts from the bottom to the top (five parts of cinnamon bark in Hetai Town) and dried in the indoor shade.

### 2.2 Investigation for germplasm resources of *C. cassia*

First, the distribution and occurrence of *C. cassia* in Guangdong and Guangxi were obtained by referring to the literature and searching for internet information (Xu et al., 2004; Wei et al., 2006; Lu et al., 2010; Yang et al., 2013). Gaoyao County, Deqing County, Luoding County, Fangchenggang City, Yulin City, and Guiping City were selected as the field investigation sites. The population sites in this survey included 3 districts in Guangdong and 4 districts in Guangxi, and latitudes and longitudes were located between N22°63’–N23°35’ and E108°01’–E112°25’, respectively. The range of annual mean temperature and precipitation in the seven regions in 2022 were 21.5–22.3 °and 1513.0–2690.0 mm, and the range of elevation was 114–493 m, which were well-suited for the growth of *C. Cassia* (**Supplementary Table S1**). *C. cassia* field survey was carried out by investigating nine important datasets: source, germplasm resource type, latitude and longitude, elevation, annual average temperature, annual average precipitation, height of the original tree, trunk girth of the original tree, and tree age of the original tree. Simultaneously, we measured the height, leaf length, leaf width, and stem diameter of the one-year-old *C. cassia* seedlings. Leaf length and width of the tenth leaf of one-year-old *C. cassia* seedlings were measured from bottom to top. Ten seedlings were randomly selected from each sample for measurements. The phenotypic characteristics of *C. cassia* accessions were measured using a Vernier caliper and tapeline, and the latitude and longitude were determined using a global position system navigator. The meteorological data constituted the annual average meteorological data published by the local meteorological bureau.

### 2.3 Determination of moisture, water-soluble extract, and volatile oil contents

The moisture, water-soluble extract, and volatile oil contents of *C. Cassia* were determined according to the determination methods (No.0832, No.2201, and No.2204) from the general rules of the CH.P, 2020 edition. The *C. cassia* samples were ground to a powder and passed through a 40-mesh sieve to obtain a finer powder. Fifty grams of *C. cassia* powder, which was precisely weighed and 10 times the volume of distilled water, was placed in a round-bottom flask and soaked for 1 h. The water vapor reflux method was used for reflux extraction for 5 h until there were no obvious oil droplets in the effluent. The cooled effluent was dried over anhydrous sodium sulfate to obtain the volatile oil from *C. cassia*.

Regarding data processing, Spearman correlation analysis using SPSS 28.0 software was performed on seven morphological parameters of cinnamon (height, trunk girth, tree age of the original tree, height, leaf length, leaf width, and stem diameter of the one-year-old *C. Cassia* seedlings), elevation, latitude and longitude, and volatile oil content.

### 2.4 Determination of volatile oil and cinnamaldehyde in cassia bark of different thickness

Chromatographic analysis was performed using HPLC (Unimicro Easy SepTM-1020LC, US). The column was C18 (250 mm × 4.6 m, 5 μm, Ecosil, USA). The mobile phase consisted of acetonitrile (A) and -0.1% phosphoric acid aqueous solution (B), and the linear gradient was set as follows: 0–155 min for 32% A to 45% A, 15–21 min for 45% A to 50% A, and 21–26 min for 50% A. Additionally, a volume flow of 1.0 mL·min^−1^, sample injection volume of 20 μL, room temperature for the column, and detection wavelength of 260 nm were established (Wu et al., 2019).

The standards for volatile oil components, namely cinnamaldehyde, cinnamyl alcohol, cinnamic acid, and 2-methoxycinnamaldehyde were purchased from Shanghai Yuanye Biotechnology Co., LTD (Shanghai, China). The purity of the standards was higher than 98%, and their lot numbers were B21081, B21080, B21082, and B27438, respectively. Following sample crushing, 0.2 g of each sample was soaked in 25 mL of methanol and weighed. After a half-hour ultrasonic extraction, the sample was weighed, and the lost mass was compensated with methanol. After collecting and filtering the upper layer using a 0.22 μm microporous membrane filter, it was transferred for high-performance liquid chromatography (HPLC) analysis (Wu et al., 2019).

### 2.5 DNA extraction and sequencing

Approximately 100 mg of fresh stem tissue was placed in a mortar and ground with liquid nitrogen. The ground tissue was transferred into a 1.5 mL centrifuge tube for genomic DNA extraction. Genomic DNA was extracted using the FastPure Plant DNA Isolation Mini Kit. Kit Manufacturer: Nanjing Vazyme Biotech Co., Ltd., China; reagent box type: DC104. The extracted genomic DNA was examined and stored at -20°C. DNA integrity was ascertained using an Agilent 2100 Bioanalyzer, and the quality and concentration of the DNA samples were evaluated using a Nanodrop spectrophotometer (Thermo Scientific, USA).

The VAHTS Universal DNA Library Prep Kit for Illumina V3 (Nanjing Vazyme Biotech Co., Ltd., China) was used to construct a paired-end sequencing library with fragment size of 350 bp from qualified samples. After the library was constructed, Agilent 2100 Bioanalyzer (Agilent Technologies,USA) were used for quality control. An Illumina NovaSeq 6000 high-throughput sequencing platform (Illumina, USA) was used for DNA library sequencing. The PE150 (pin-end, 150) sequencing strategy was used. Illumina high-throughput sequencing results were initially presented as raw image data files, which were converted into raw reads after base calling using the CASAVA software. High-quality clean reads were obtained from the original sequence using Fastp v0.20.1 software.

### 2.6 SNP calling

Illumina high-throughput sequencing results were initially presented as image data files, which were converted into raw reads after base calling by CASAVA software (https://www.britannica.com/plant/cassava). High quality clean reads were obtained by using Fastp v0.20.1 software (https://github.com/OpenGene/fastp) with the default parameters for data quality control of the original sequences (**Supplementary Table S3**).

The BWA software (https://github.com/lh3/bwa) was used to align the clean reads of 71 C*. cassia* accessions to the reference genome sequence using the default settings (Dobin et al., 2013). To reduce SNP detection errors caused by alignment errors, sequencing fragments that were compared to the SNP region were double-ended and simultaneously aligned to the reference sequence (accession number: CNA0140271).

Based on the comparison results, deepvariant software (version 1.3.0, https://github.com/google/deepvariant) was used to detect the SNPs (Poplin et al., 2018; Eriksson et al., 2022), and samtools-mpileup and Python programs were used to test for SNP genotypes, base sequencing quality, and read comparison quality for the comprehensive identification of SNP polymorphic sites.

### 2.7 SNP genetic diversity analysis

The Shannon-Weaver (H′) index, minor allele frequency (MAF), observed heterozygosity (Ho), expected heterozygosity (He), observed number of alleles (Na), effective number of alleles (Ne), Shannon’s information index (I), percentage of polymorphic bands (PPB), genetic identity (GI), and genetic diversity (GD) are examples of the parameters used to estimate genetic diversity in populations. These parameters were estimated using PowerMarker 3.25 and Popgene version 1.32 software (Danecek et al., 2011). In this study, VCFtools software (version 0.1.16) was used to convert SNP information into a format recognizable by the PLINK software version v1.90 (http://pngu.mgh.harvard.edu/purcell/plink/) (Purcell et al., 2007). Ho, He, MAF, and polymorphism information content (PIC) were calculated using PIC_CALC 0.6 (Nagy et al., 2012).

Analysis of molecular variance (AMOVA) using the GeneAlEx 6.502 tool with 1000 permutations was used to characterize the variance components of *C. Cassia* individuals and population differentiation among the seven postulated subgroups (Peakall and Smouse, 2012).

### 2.8 Population structure analysis

The ADMIXTURE (Version 1.3.0) software (https://dalexander.github.io/admixture) was used to analyze the population structure (Pritchard et al., 2000). The obtained SNP information is converted into a binary PLINK file that is recognized by ADMIXTURE software. The K value is calculated using ADMIXTURE. Set the K value to 1–24, and theoretically select the K value with the smallest CV error (Cross-validation error) as the best clustering (Perez et al., 2021).

### 2.9 Phylogenetic and principal component analysis

The ML phylogenetic tree of *C. cassia* was analyzed using the optimal model GTR+F+R5 implemented in IQ-TREE software (version 2.0.5) (Alexander et al., 2009). The optimal model selection was based on Bayesian Information Criterion (BIC) scores. To provide more evidence for the number and composition of populations of *C. cassia* accessions, this study also performed principal component analysis (PCA) using the default settings of the Genome-wide Complex-Trait Analysis (GCTA) software (Zheng et al., 2013).

## 3 Results

### 3.1 Analysis of morphological traits of *C. cassia*

**Figure 2:**
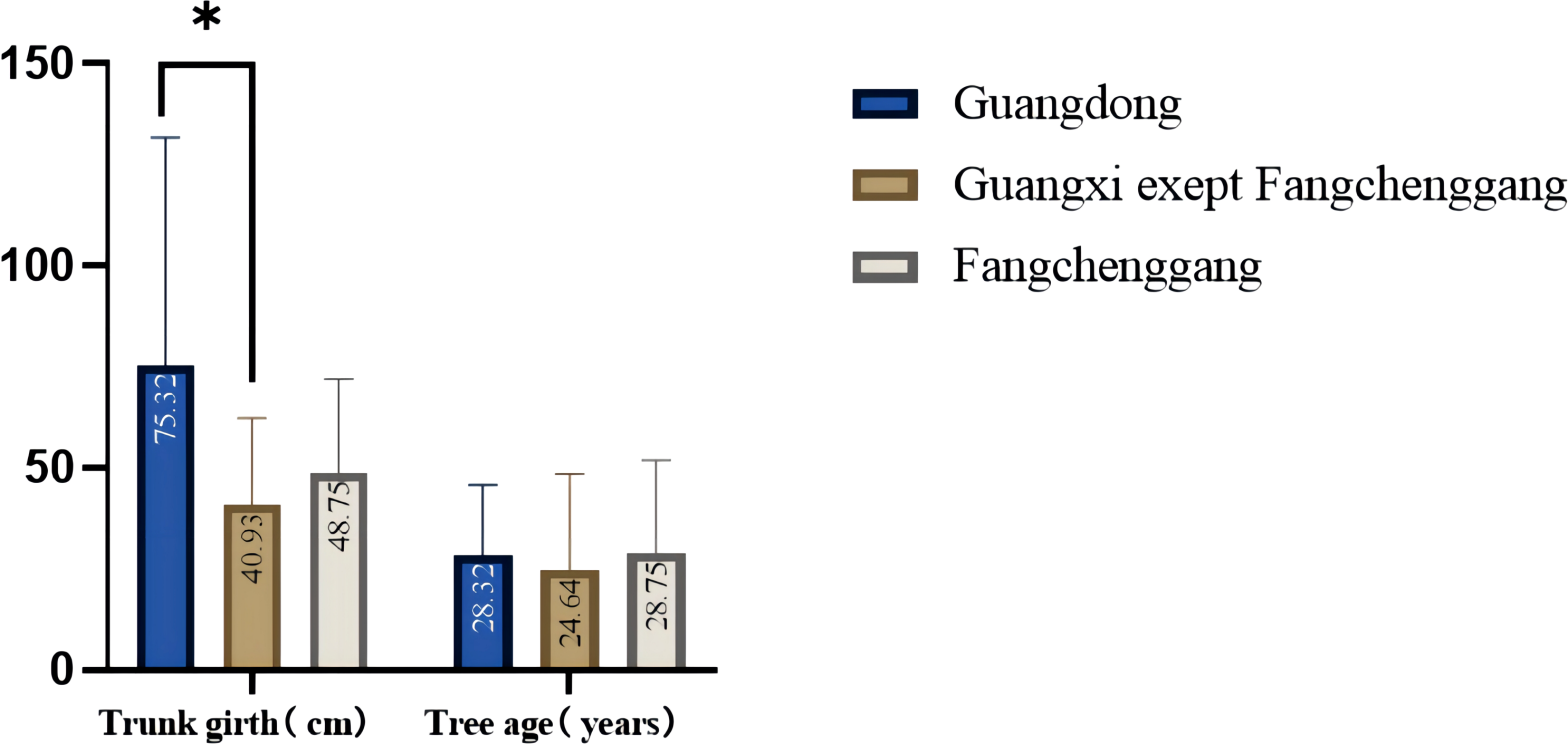
Comparison of trunk girth and age of the original trees in Guangdong and Guangxi (*p<0.05; *p<0.01; ***p<0.001; one-way ANOVA).

The phenotypic characteristics of *C. cassia* in different populations showed some differences (**Figures 1B, C**). **Table 1** and **Supplementary Table S4** show the variations in the primary morphological characteristics of *C. cassia* from several accessions. In this study, the original *C. cassia* trees were between 6 and 25 m tall, 35 to 373 cm thick, and 8 to 100 years old; 40 samples were wild and the rest were cultivated. In particular, 37 wild *C. cassia* samples, which generally had longer growth years and thicker trunks, were sourced from Guangdong, and the other three were sourced from Guangxi. We analyzed the trunk girth and age of the original trees. The average trunk girth and tree age of the original tree from Guangdong were 75.32 cm and 28.32 years, respectively, which were higher than those from Guangxi (40.93 cm and 24.64 years, respectively). The trees in Fangchenggang were older than those in the other areas (**Figure 2**).

**Table 1:** Morphological traits of *C. cassia* germplasm utilized in this study.

Variables	Max	Min	Mean	SD	CV(%)
Height of the original tree (m)	25.00	4.00	11.30	3.90	34.79
Trunk girth of the original tree (cm)	373.00	11.00	67.05	51.81	77.27
Age of the original tree (years)	100.00	3.00	27.62	18.86	68.27
Height of the one-year-old *C. cassia* seedlings (cm)	191.93	70.10	141.78	27.30	19.26
Leaf length of the one-year-old *C. cassia* seedlings (cm)	39.80	13.00	22.65	4.30	18.98
Leaf width of the one-year-old *C. cassia* seedlings (cm)	23.80	5.77	8.12	3.18	39.16
Stem diameter of the one-year-old *C. Cassia* seedlings (cm)	1.50	0.13	0.73	0.33	45.21

SD was calculated based on the measured values of the nine traits. CV was estimated as the ratio of the standard deviation to the mean of all accessions. SD, standard deviation; CV, coefficient of variation.

**Figure 3:**
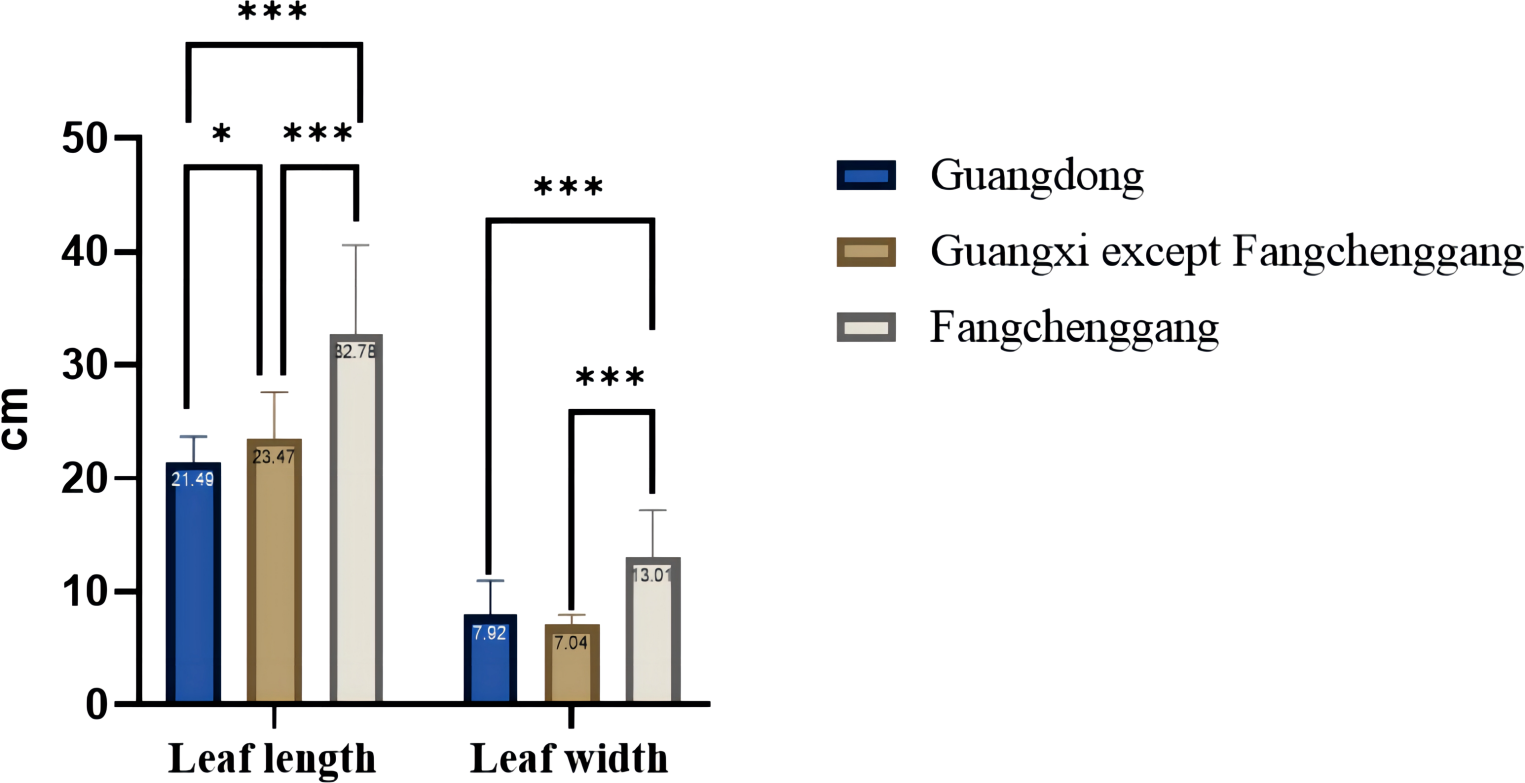
Comparison of leaf length and leaf width from the one-year-old *C. cassia* seedlings in Guangdong and Guangxi (*p<0.05; *p<0.01; ***p<0.001; one-way ANOVA).

The height of the one-year-old *C. cassia* seedlings ranged from 70.10 cm to a maximum of 191.93 cm (RG52). The leaf length of the one-year-old *C. cassia* seedlings ranged from 13.00 cm to a maximum of 39.80 cm (RG70). The leaf width of the one-year-old *C. cassia* seedlings ranged from 5.77 cm to a maximum of 23.80 cm (RG11). The range of the stem diameter for the one-year-old *C. cassia* seedlings was 0.13–1.50 cm, with RG68 showing the largest stem diameter (1.50 cm). The average leaf length and width of the one-year-old *C. cassia* seedlings from Fangchenggang were 32.78 and 13.01 cm, respectively, which were higher than those from Guangdong (21.49 and 7.92 cm, respectively) and other areas of Guangxi (23.47 and 7.04 cm, respectively) (**Figure 3**). There were no significant differences in leaf length or width between Guangdong and Guangxi, except for Fangchenggang. These findings indicate that the Guangxi germplasm had short development years and fleshy, thick, and relatively wide leaves, particularly Fangchenggang, whereas Guangdong samples generally had lengthy growth years and towering trees with strong trunks.

The coefficient of variation (CV) of the phenotypic traits was computed to evaluate the genetic diversity of the *C. cassia* accessions. With an average of 43.28%, the CVs of the seven attributes ranged from 18.98% to 77.27%. The most variable trait was trunk girth of the original tree (77.27%), followed by tree-age of the original tree (68.27%), stem diameter of the one-year-old *C. cassia* seedlings (45.21%), and leaf width of the one-year-old *C. cassia* seedlings (39.16%), while the leaf length of the one-year-old *C. cassia* seedlings showed the least variation (18.98%), indicating that the tree age varied greatly and the leaf length was almost uniform among different accessions. The majority of the seven phenotypic variables showed CV higher than 20%, indicating clear variability of these features (**Table 1**).

The morphology of wild species varies greatly across populations. For example, leaves from Guiping and Fangchenggang (Guangxi) are thick, meaty, and somewhat wide. The seeds are black-purple in color and have shiny surfaces. In addition, we found a type of *C. cassia* with purple volatile oil, strong fragrance, and wider leaves. The local called “Zi You Gui” has larger planting area. In Fangchenggang, we discovered a unique wild plant with extremely large, glabrous leaves and black-purple seeds. The average leaf length and width were 35.00 cm and 15.20 cm, respectively.

### 3.2 Analysis of moisture, water-soluble extract, and volatile oil contents of *C. cassia*

The moisture, water-soluble extract, and volatile oil contents of *C. cassia* are shown in **Supplementary Figure S1** and **Supplementary Table S5**. The findings demonstrated wide variations in the volatile oil concentrations of *C. cassia* from various sources. The average amount of volatile oils in each sample was 2.3% and ranged from 0.5% to 6.3%. RG37 contained the highest amount of volatile oils, whereas RG07 contained the lowest. The average volatile oil contents in the seven districts were 1.7%, 2.0%, 2.5%, 3.2%, 3.4%, 3.2%, and 3.6% for Deqing, Gaoyao, Luoding, Guiping, Pingnan, Yulin and Fangchenggang, respectively. The germplasm from Fangchenggang had the highest volatile oil concentration, followed by that from Pingnan, whereas the germplasm from Deqing had the lowest volatile oil concentration. Each sample had a different moisture content ranging from 10.3% to 18.0% on average, with RG52 having the highest moisture content and RG08 having the lowest (**Table 2**). The average moisture contents in the seven districts were 14.2%, 14.0%, 14.7%,13.5%,15.2%, 14.75%, and 14.3% for Deqing, Gaoyao, Luoding, Guiping, Pingnan, Yulin and Fangchenggang, respectively. The germplasm from Guiping had the lowest moisture content, whereas that from Pingnan had the highest moisture content, followed by that from Yulin. The water-soluble extract content of each sample ranged from 9.9% to 23.0% with an average of 18.0%. RG46 had the highest water-soluble extract concentration, while RG16 had the lowest. The average water-soluble extract contents in the seven districts were 18.1%, 17.4%, 18.9%, 18.5%, 17.8%, 18.4%, and 18.9% for Deqing, Gaoyao, Luoding, Guiping, Pingnan, Yulin and Fangchenggang, respectively. The most water-soluble extract was found in the germplasms of Fangchenggang, whereas the least was found in Yulin and Gaoyao.

**Table 2:** Variation of volatile oil contents of *C. cassia* (%).

Population	Max	Min	Mean	SD	CV(%)
G1 (Deqing)	3.10	1.00	1.70	0.61	0.35
G2 (Gaoyao)	3.90	0.50	2.00	0.88	0.44
G3 (Luoding)	6.30	1.20	2.50	1.40	0.57
G4 (Guiping)	4.40	1.90	3.20	0.86	0.27
G5 (Pingnan)	4.70	2.70	3.30	0.73	0.22
G6 (Yulin)	3.20	3.10	3.15	0.05	0.02
G7 (Fangchenggang)	4.00	2.90	3.60	0.43	0.12

SD, standard deviation; CV, coefficient of variation.

**Figure 4:**
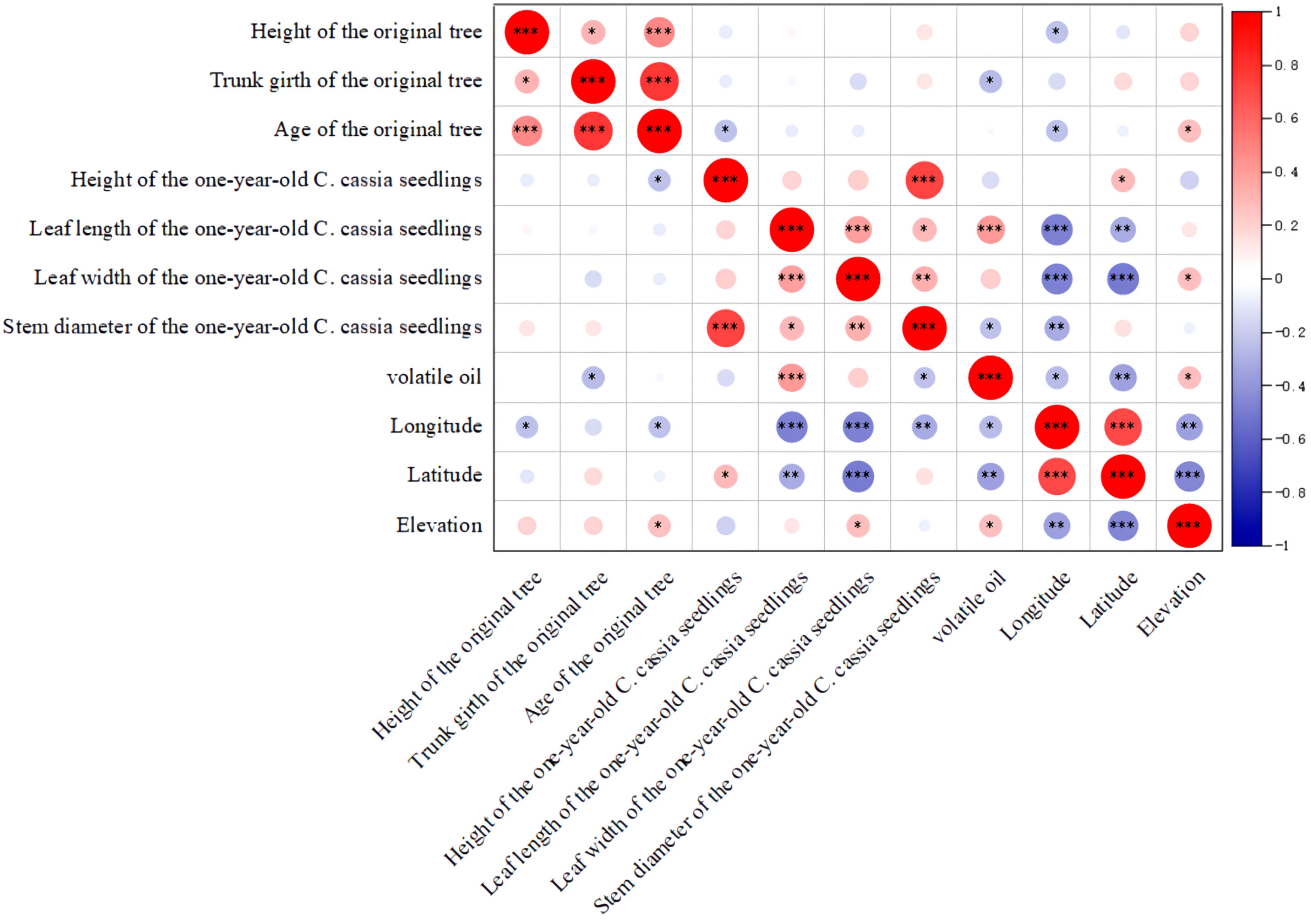
Correlation analysis of morphology and chemical constituents of *C. cassia* (L.) J.Presl (*p<0.05, **p<0.01, ***p<0.001, Pearson correlation analysis).

To investigate the connection between the phenotypic qualities, chemical components, and geographic location, we performed a Spearman correlation analysis. The results showed that the height of the one-year-old *C. cassia* seedlings was positively correlated with stem diameter, indicating that the higher the tree, the larger the diameter (**Figure 4**; **Supplementary Table S6**). The age of the original tree was also positively correlated with tree height and tree trunk girth, which is consistent with traditional grading standards. Interestingly, we found that three phenotypic characteristics were correlated with volatile oil content, i.e. samples with longer and broader leaves and originating from higher elevations showed higher essential oil content. This provides a scientific foundation for the logical cultivation of *C. cassia*, suggesting that these traits can be used to create new types with highly active components. However, in contrast to the conventional grading criteria, we discovered a negative association between tree diameter and volatile oil concentration, indicating that samples with a smaller tree diameter had a higher volatile oil content. Furthermore, a negative correlation was observed between latitude and longitude and phenotypic traits such as leaf width, leaf length, tree height, tree diameter, and volatile oil content. These findings indicate that plantations at lower latitudes and longitudes are better suited for the growth and development of *C. cassia*.

**Figure 5:**
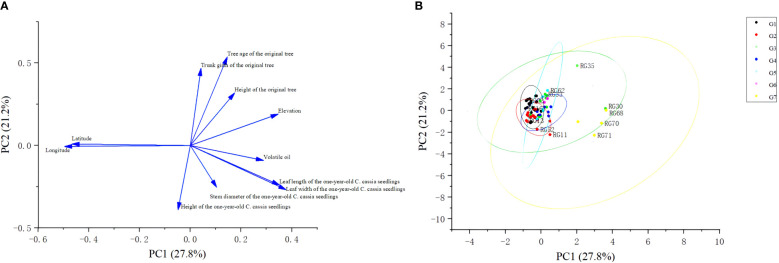
Principal component analysis of variables. **(A)** Loading plot of 71 *C. cassia* samples, geographical variables include longitude, latitude and altitude, Chemical composition variable include volatile oil, other variables are morphological indicators. **(B)** Score plot of 71 *C. cassia* samples.

Additionally, The PCA analysis showed that 75.853% of the diversity was explained by the first four main components (**Figure 5A**; **Supplementary Table S7**). With features such as leaf length and leaf leaf width of one-year-old *C. cassia* seedlings, elevation, and volatile oil, PC1 with an eigenvalue of 3.054 accounted for 27.767% of the total variation. The height, trunk girth, and age of the original tree contributed remarkably to PC2, accounting for 21.194% of the variation. The stem diameter and height of one-year-old *C. cassia* seedlings were among the variables for which PC3 accounted for 18.812% of the total variation. All 11 variables were slightly differentiated and could be used to discriminate *C. cassia* accessions. In addition, we also conducted PCA analysis on 71 samples based on these 11 indices, and the results are shown in **Figure 5B**. The results showed that RG30, RG68, RG69, RG70 and RG71 clustered together, and the rest of the samples clustered together.

### 3.3 Changes of volatile oil and cinnamaldehyde contents in cassia bark with different thickness

In a previous study, when determining the content of volatile oils in cassia bark, our group found that the cinnamaldehyde content in different samples of cinnamon medicinal materials purchased from the same batch was notably different and that the volatile oil contentbetween different thicknesses from the same tree was irregular, indicating that the quality of the cinnamon medicinal materials was uneven. Although all medicinal compounds of cinnamon are derived from the bark, cinnamon is a tall perennial tree with a long growth period, which is affected by many environmental factors during the growth process, and the distribution of secondary metabolites in the bark may be uneven. Therefore, it is speculated that the content of cinnamaldehyde in the upper, middle, and lower parts of cassia bark may be different.

**Figure 6:**
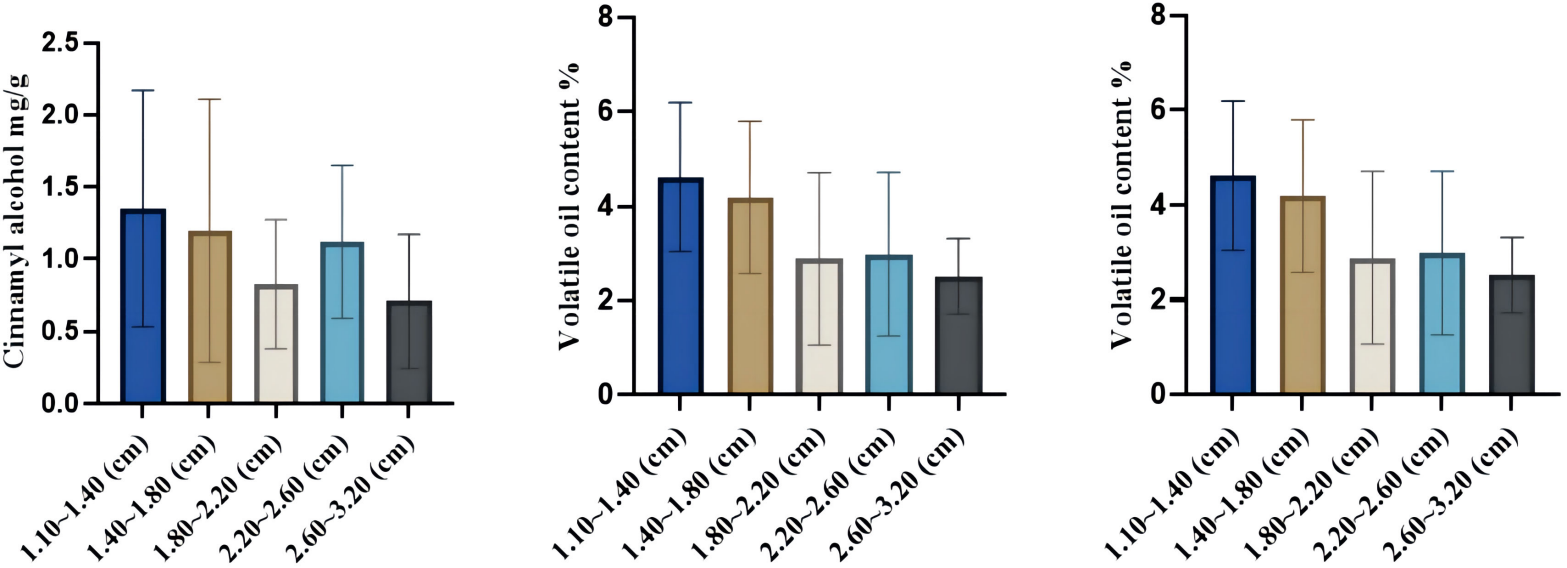
Changes of cinnamaldehyde, cinnamic acid and volatile oil contents incinnamon bark of different thicknesses.

**Figure 7:**
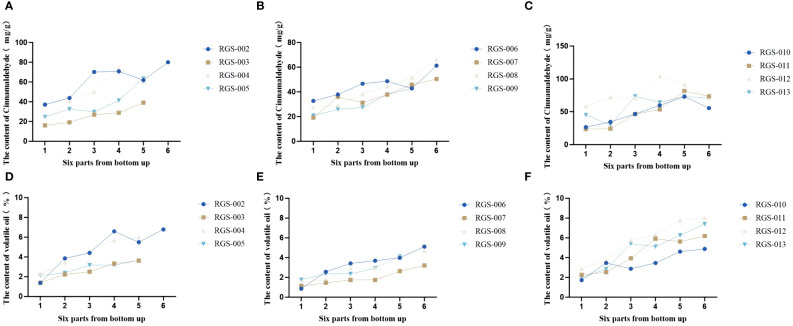
Changes of cinnamaldehyde and volatile oil contents from bottom to top in the bark of the same cinnamon tree. **(A–C)** The cinnamaldehyde content in cinnamon samples from Hetai Town, Gaoliang Town, Tanbin Town, Guangdong Province. **(D–F)** The volatile oilcontent in cinnamon samples from Hetai Town, Gaoliang Town, Tanbin Town, Guangdong Province.

In this study, HPLC was used to determine the total amount of volatile oil and the difference in the content of four volatile oil components–cinnamaldehyde, cinnamic acid, cinnamyl alcohol, and 2-methoxycinnamaldehyde–in the upper, middle, and lower parts of the *C. cassia* tree. The results showed that the contents of cinnamaldehyde, cinnamyl alcohol, cinnamic acid, and 2-methoxycinnamaldehyde in the 12 cassia bark samples were 16.10–104.10, 0.16–3.52, 0.18–1.06, and 0–10.24 mg/g, respectively. The mean values were 48.19, 1.08, 0.51 and 1.96 mg/g, respectively. The volatile oil content ranged from 0.85% to 8.05% with an average of 3.70% (**Supplementary Table S2**). Interestingly, the cinnamaldehyde and cinnamyl alcohol contents first decreased and then increased with increasing cassia bark thickness. Cinnamaldehyde content was highest when the thickness of the cassia bark was 2.60–3.20 mm (up to 44.10 mg/g), while the content of cinnamaldehyde was lowest when the thickness of the cassia bark was 2.20–2.60 mm (up to 36.86 mg/g). The cinnamyl alcohol content was highest when the thickness of the cassia bark was 1.10–1.40 mm (1.35 mg/g), while cinnamaldehyde content was at its lowest (0.83 mg/g) when the thickness of the cassia bark was 1.80–2.20 mm. The cinnamic acid content of the 12 cassia bark samples first increased and then decreased with increasing cassia bark thickness. The content of cinnamic acid was at its highest (0.57 mg/g) when the thickness of the cassia bark was between 1.40 and 1.80 mm. The volatile oil content decreased with an increase in the thickness of the cassia bark, and the content was highest when the thickness of the cassia bark was between 1.10 and 1.40 mm (**Figure 6**; **Supplementary Figure S2**). In the same cinnamon tree, the cinnamaldehyde and volatile oil contents were negatively correlated with cross-sectional thickness; that is, the cinnamaldehyde and volatile oil contents increased from the near-ground part to the upper part of each cassia bark sample (**Figure 7**). The cinnamaldehyde and volatile oil contents in the upper, middle, and lower parts of the cassia bark were markedly different; the difference in cinnamaldehyde content in the same tree was up to two times; and the difference in volatile oil was up to six times, suggesting that this difference might be one of the reasons for the uneven quality of the cassia bark.

### 3.4 SNP markers quality and diversity

**Figure 8:**
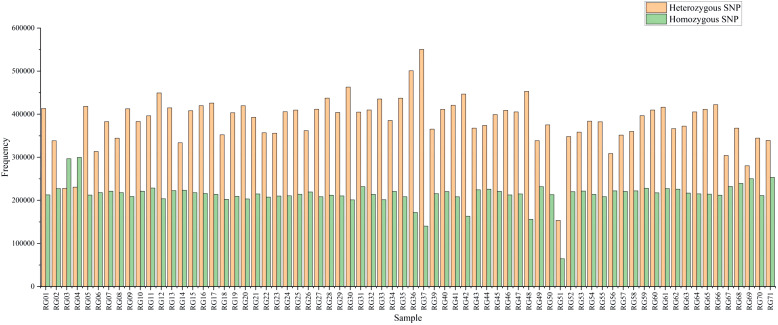
The SNP number in 71 *C. cassia* samples.

**Figure 9:**
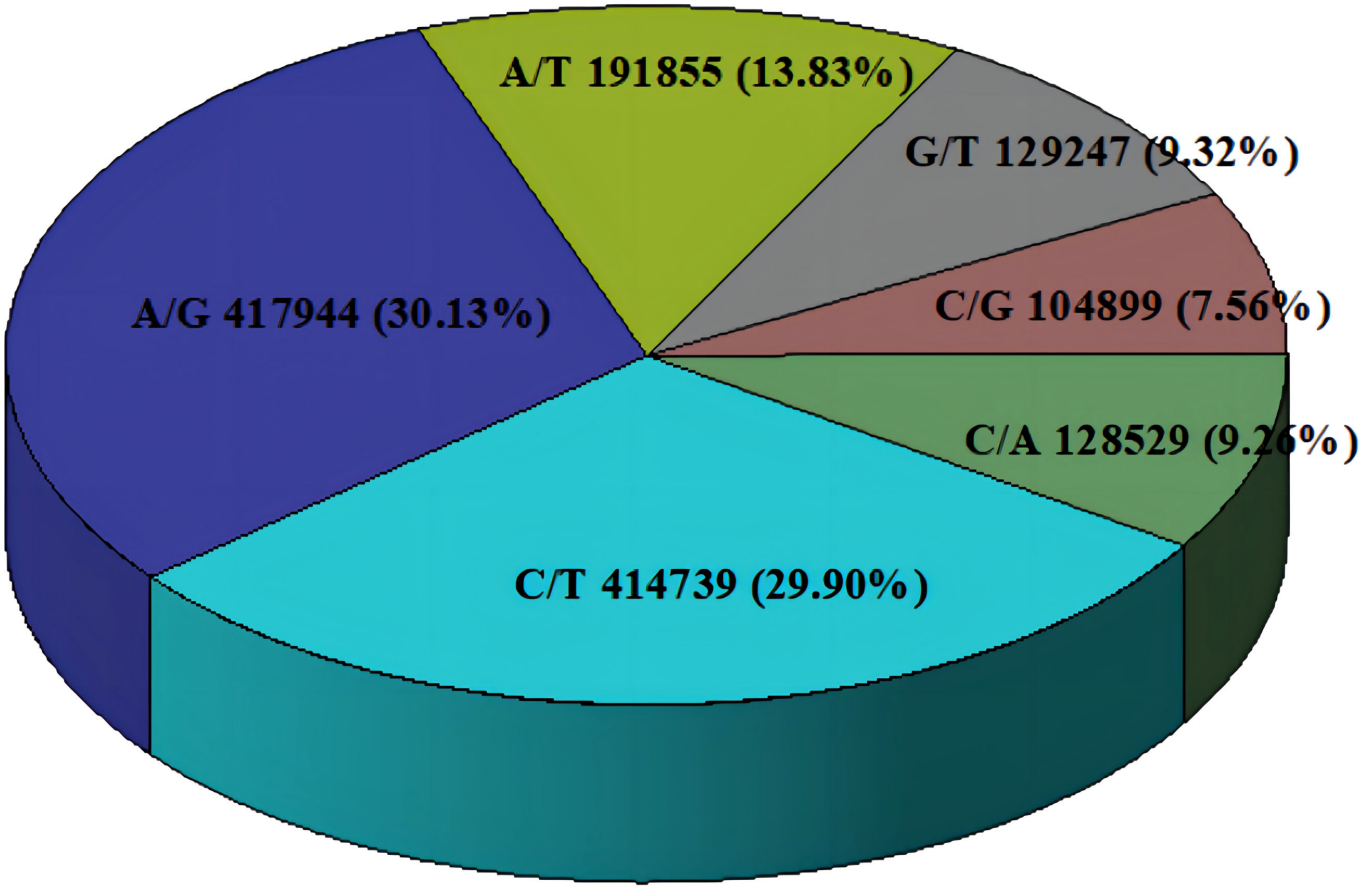
SNP mutation types.

A total of 1,387,213 SNP markers were used in subsequent analyses when the identified SNPs of all 71 C*. cassia* samples were combined. SNP markers included across all libraries were screened for non-biallelic sites, sites with MAF < 0.05, and sites with deletion rates greater than 20%. In contrast, 1,387,213 SNP markers were sufficient to estimate the genetic diversity and population structure of *C. cassia*. The average number of SNPs per sample was 5598,397, with a range of 218,134–6690,682. The sample with the highest number of SNPs was RG37, whereas the sample with the lowest number was RG51. All 71 accessions had 15,014,708 homozygous and 26,863,047 heterozygous SNPs, representing 35.87% and 64.13% of all the SNPs, respectively. There was no discernible difference between the wild (64.15%) and cultivated (63.31%) samples, with an average heterozygosity rate of 63.93% (**Figure 8**; **Supplementary Table S8**). The two most common types of substitutions in the SNP dataset were transversions (C/A, 9.26%; G/T, 9.32%; C/G, 7.56%; A/T, 13.83%) and transitions (C/T, 29.90%; A/G, 30.13%). These substitutions included 832,683 (60.01%) and 554,530 (39.99%) SNPs, respectively (**Figure 9**; **Supplementary Table S9**). Heterozygosity is considered the best metric for assessing the genetic diversity of a population because it can represent the genetic variance of the population at several loci. A population’s genetic diversity can be measured using Ho; the higher the Ho, the more diverse the population’s genetic makeup. With an average of 0.32 and 0.49, respectively, Ho and He ranged from 0.14 to 0.44 and 0.18 to 0.92, respectively, indicating that *C. Cassia* populations were less impacted by inbreeding, artificial selection, and other factors and were in a state of genetic balance.

The Luoding population had the greatest Na at 1.919, while the Yulin population (1.655) had the lowest value. In the Yulin population, the Ne varied from 1.545 to 1.610 in the Gaoyao population. With a mean of 0.478, the Shannon’s information index (I) ranged from 0.418 (Yulin) to 0.513 (Gaoyao), indicating a comparatively high level of community diversity in Gaoyao. The PPB percentage varied between Yulin (65.52%) and Deqing (98.88%) with an average of 86.94%. In contrast, the Ho and He ranged from 0.2 to 0.4 and 0.4 to 0.5, indicating the largest number of SNP loci (464,901 and 611,493, respectively), followed by 0 to 0.2 and 0.1 to 0.2 (**Table 3**). As seen in **Supplementary Figure S3**, the MAF distribution was examined. The highest number of SNP loci (approximately 320,000) in MAF ranged from 0.05 to 0.10, followed by 0.10 to 0.15, suggesting that the *C. cassia* populations under investigation had a low level of genetic diversity. The polymorphism height of the molecular markers was quantified using the PIC metric. It is generally believed that 0.25 < PIC < 0.5 represents a moderate polymorphism site, PIC > 0.5 represents a high polymorphism site, and PIC < 0.25 represents a low polymorphism site (Wang et al., 2019). The PIC varied from 0.075 to 0.375, and the highest number of SNPs (approximately 550,000) was between 0.300–0.375 and 0.225–0.300, respectively. These results suggest that the *C. cassia* populations under investigation had a moderate degree of genetic variation (**Supplementary Figure S4**).

**Table 3:** Summary statistics of molecular diversity revealed by SNP markers in Seven *C. cassia* populations from two provinces in China.

Population	N	Na	Ne	I	Ho	He	PPB
G1 (Deqing)	21	1.989	1.605	0.510	0.489	0.342	98.88%
G2 (Gaoyao)	22	1.988	1.610	0.513	0.479	0.345	98.76%
G3 (Luoding)	9	1.919	1.601	0.496	0.499	0.336	91.88%
G4 (Guiping)	7	1.890	1.588	0.487	0.478	0.330	88.96%
G5 (Pingnan)	5	1.833	1.583	0.475	0.485	0.324	83.34%
G6 (Yulin)	2	1.655	1.545	0.418	0.470	0.293	65.52%
G7 (Fangchenggang)	4	1.750	1.559	0.445	0.466	0.307	74.96%
Mean	10	1.860	1.584	0.478	0.481	0.325	86.04%

Na, Observed number of alleles; Ne, Effective number of alleles; I, Shannon’s information index; Ho, Observed heterozygosity; He, Expected heterozygosity; PPB, Percentage of polymorphic bands.

AMOVA, which can yield important information, was performed using a model-based analysis to assess the population’s genetic constitution based on its consistency and reliability (**Table 4**). The findings showed that individuals within populations accounted for the majority of the genetic variation (95.87%; Df = 111; sum squares = 878.895), whereas populations within groups accounted for 0.59% of the variation (Df = 3; sum squares = 26.216), and the remaining variation was found among groups. AMOVA demonstrated that individuals accounted for the majority of the genetic diversity in the *C. cassia* germplasm. In addition, research has revealed that there is little genetic variation among the seven populations, owing to gene crossovers. The genetic distances (GD) and genetic identities (GI) between seven populations were computed based on the SNP locus data. The findings indicated that the GD between Fangchenggang and the other populations were greater, ranging from 0.049 to 0.082, with an average of 0.059, whereas the GD between Deqing and Gaoyao were lowest (0.008). Furthermore, the differences between the other populations were comparatively small, averaging 0.030 and ranging from 0.014 to 0.057 (**Supplementary Table S10**). Except for Fangchenggang, there was a high degree of genetic relatedness among the *C. cassia* subpopulations, which is in line with the findings of the study of plant morphological characteristics. Fangchenggang and Yulin showed the greatest GD (0.082), whereas Deqing and Gaoyao showed the shortest GD (0.049). A mean GI of 0.965 was obtained, ranging from 0.921 to 0.992, A trend with which the GD was at odds.

**Table 4:** Analysis of molecular variance (AMOVA) in 71 C*. cassia* accessions based on SNP loci.

Source of variation	Df	Sum of squares	Variance components	Percentage of variation
Among groups	1	19.426	0.159	3.54
Among populations within groups	3	26.216	0.029	0.59
Among individuals within populations	111	878.895	4.457	95.87
Total	115	924.537	4.663	100.00

SNP, single nucleotide polymorphism; Df, degree of freedom.

### 3.5 Phylogenetic analysis of the *C. cassia* population

**Figure 10:**
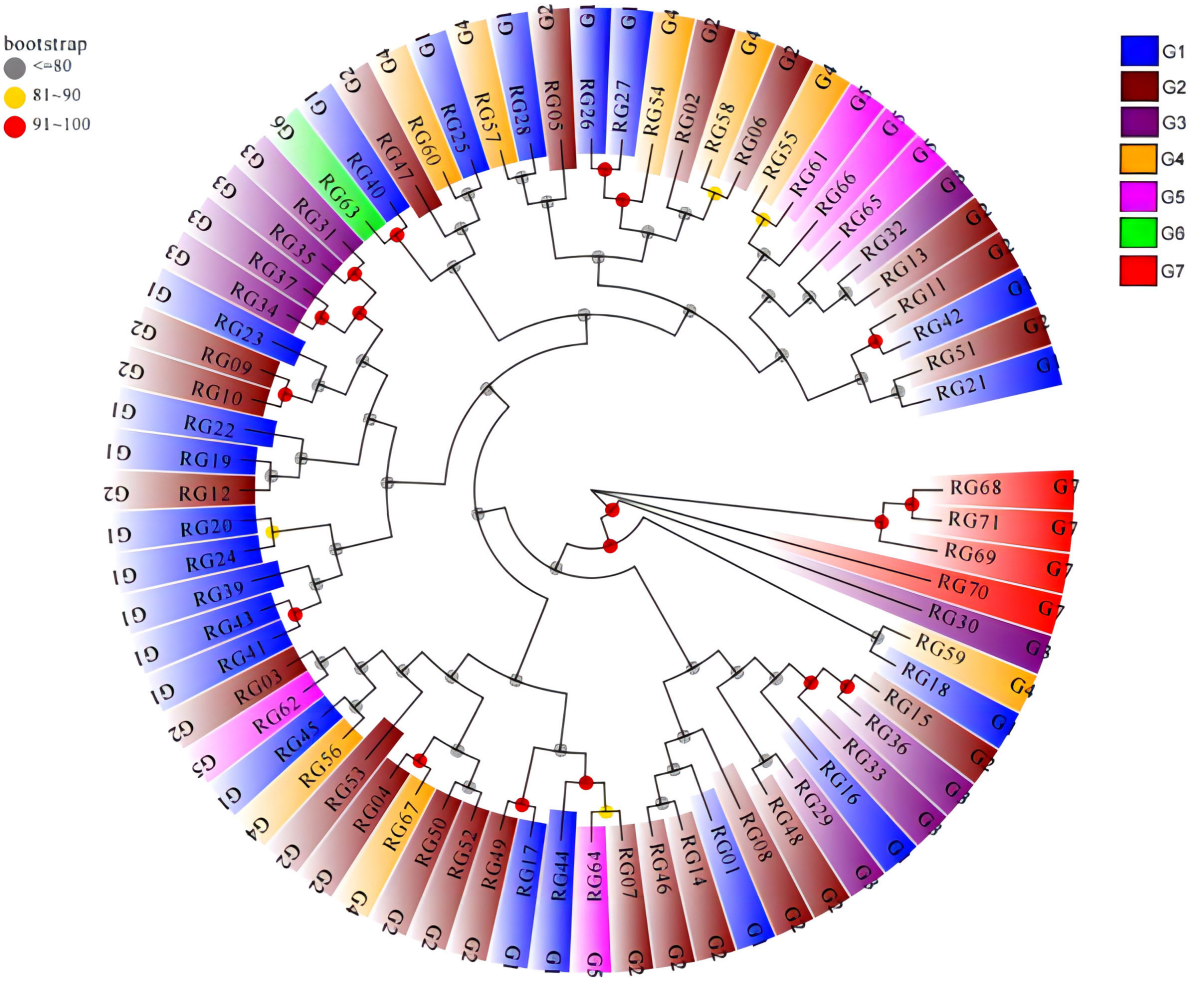
Phylogenetic tree of the 71 *C. cassia* accessions based on SNP marker. G1: Deqing, G2: Gaoyao, G3: Luoding, G4: Guiping, G5: Pingnan, G6: Yulin, G7: Fangchenggang.

Based on genetic distance, a phylogenetic tree was created using the Neighbour Joining (NJ), and the results showed that the 71 C*. cassia* accessions were mostly grouped into two clusters (**Figure 10**). Three accessions from Fangchenggang City, which included a sample of wild *C. cassia* (RG68), were included in cluster I. The genetic difference between RG68 and RG69 in this cluster was the highest at GD = 0.210, whereas the genetic distance between GX68 and GX71 was the lowest at GD = 0.197. Despite their genetic diversity, all these individuals could be clearly separated from cultivars based on their phenotypic features. Because the Fangchenggang accessions were closely linked to one another, it was possible to use them to increase the genetic background. Cluster II contained 68 accessions, most of which developed in Guangdong and Guangxi, with the exception of Fangchenggang. It contained all wild-type samples, except for RG68. The GD in this clade ranged from 0.150 to 0.306, and some accessions such as RG26 and RG27 had very little genetic distance, making them closely related (**Supplementary Table S11**). Overall, there was a strong correlation between the genetic distances between *C. cassia* accessions and their geographical origins. This indicates that most *C. cassia* germplasms could be gathered from the same or comparable origin, with a small amount of test materials mixed with other groups. Except for Fangchenggang, the accessions from Guangdong and Guangxi were closely related. In 71 samples, it was not possible to distinguish between wild and cultivated types, which is consistent with the results of the cluster analysis based on phenotypic characteristics.

### 3.6 Structure analysis of the *C. cassia* population

**Figure 11:**
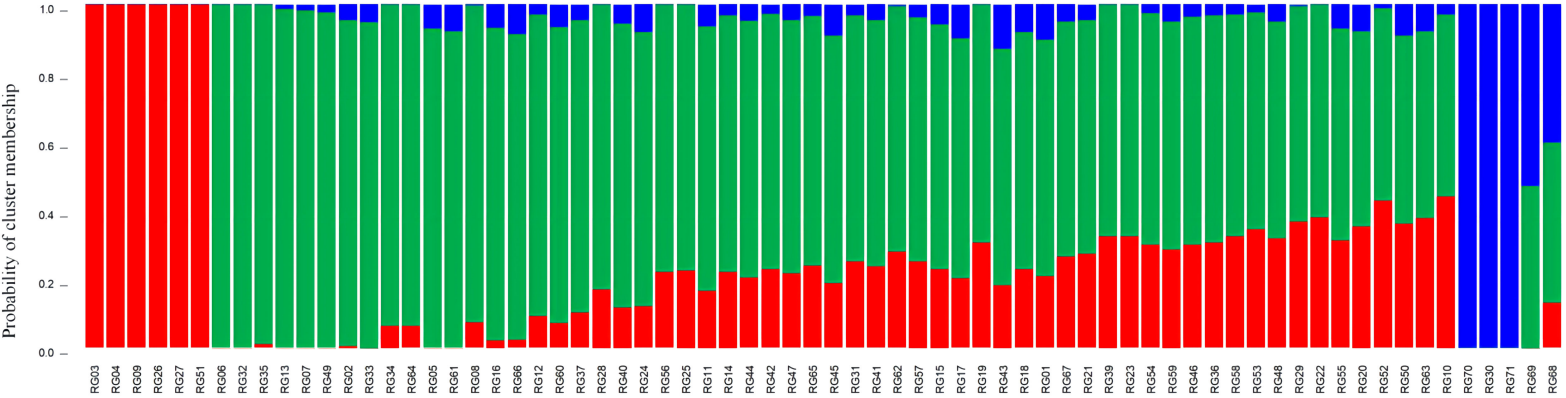
Population structure of the 71 *C. cassia* accessions.

To identify the population groups, structure analysis based on a mixed Bayesian clustering model was applied. The findings indicated that K (the number of random mating subgroups) was equal to three as the optimal number of groups (**Supplementary Figure S5**; **Supplementary Table S12**), indicating that the 71 accessions could be divided into three subpopulations (I, II, and III) (**Figure 11**). Based on shared genomic areas, the genotypes of the various populations were separated into pure and mixed types. Specifically, genotypes scoring ≥0.80 were classified as pure and split into appropriate subgroups, while genotypes scoring <0.80 were termed admixed (Luo et al., 2022). In total, 47 (or about 62%) of the 71 accessions were admixed, and the remaining 27 (approximately 38%) were pure. The admixtures showed that 75% of the genotypes were from Guangdong and 25% were from Guangxi, including three wild-types. This suggests that the genotypes from Guangxi and Guangdong have a more complex genetic background with mixed genes from multiple populations and a higher level of genetic diversity, whereas the pure genotypes from Gaoliang Town, Guangdong, and Fangchenggang City (Guangxi) have a narrower genetic background. These results are consistent with those of the phylogenetic analysis. RG70 and RG71 were pure types that originated from Fangchenggang. This suggests that they may have originated in Vietnam and might have been utilized in the breeding of *C. cassia*.

### 3.7 Principal component analysis of the *C.cassia* population

**Figure 12:**
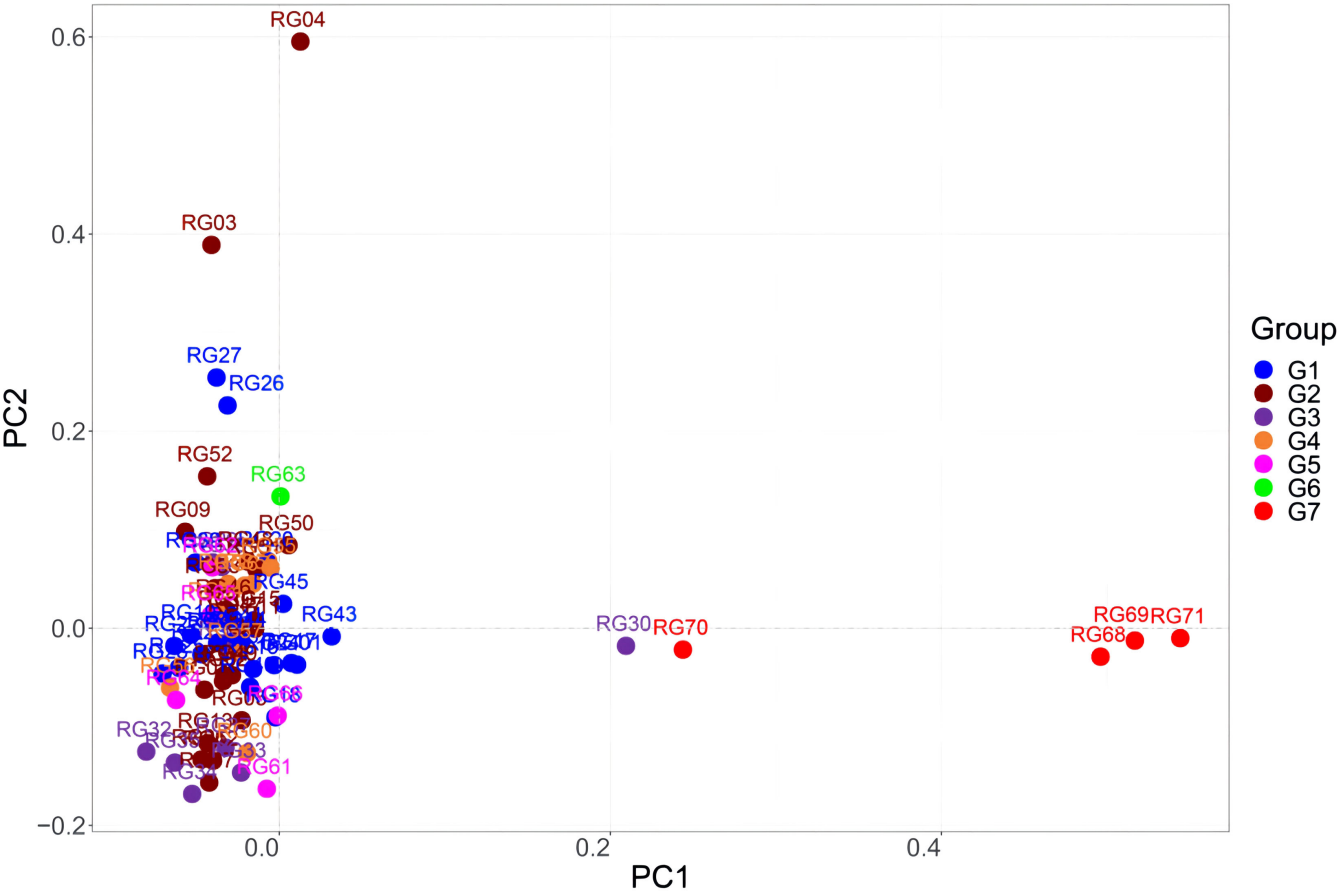
Principal component analysis (PCA) of the 71 *C. cassia* accessions. G1: Deqing, G2: Gaoyao, G3: Luoding, G4: Guiping, G5: Pingnan, G6: Yulin, G7: Fangchenggang.

The results of the PCA, which revealed three separate clusters, agreed with those of the population structure analysis. The data shown in **Figure 12** indicate that the Guangxi accessions were more dispersed than the Guangdong accessions. Except for RG30, RG68, RG69, RG70, and RG71, the Guangdong and Guangxi accessions were clustered together. This is consistent with PCA analysis results of 11 indicators. This suggests that there was low genetic variation among the germplasm lines and that there was only a small genetic divergence of genotypes from Guangdong and Guangxi. Interestingly, accessions from Fangchenggang in Guangxi were unique. One possible explanation for this could be the introduction of dispersed germplasms from other areas.

## 4 Discussion

The total genetic variety among individuals within a species or population is referred to as genetic diversity (Pyne et al., 2018; Verma et al., 2019). The study of genetic variation can be used to improve breeding plans for medicinal plants by choosing parents with superior genotypes and providing information on the degree of genetic organization within populations (Yang et al., 2018; Niu et al., 2019). Based on phenotypic and chemical composition and SNP markers, we examined the genetic diversity of *C. cassia* samples in this study. These findings demonstrated that populations of *C. cassia* exhibited a low degree of genetic variation.

### 4.1 Variations based on morphological traits

In this study, we analyzed 11 indicators of cultivated and wild *C. cassia* samples, including leaf length, leaf width, tree height, tree age, elevation, longitude, and latitude. These findings demonstrate that the *C. cassia* population’s phenotypic features exhibited a high degree of genetic variation. Variations in *C. cassia* differed greatly in phenotypic traits. For example, Fangchenggang, a variety of Guangxi, has long and broad leaves, whereas Guangdong has very narrow leaves. We found that Guangdong samples generally had long growth years and tall trees with strong trunks, whereas Guangxi germplasm had short growth years and fleshy, thick, and relatively wide leaves, especially in Fangchenggang. In our previous study, we found that the *C. cassia* planting area in Guangdong Province accounted for more than 30% of the country’s total *C. cassia* planting area. The Xijiang River Basin in Zhaoqing City, Guangdong Province, is suitable for *C. cassia* growth under the geographical environment, soil, soil quality, water quality, climate, and light conditions, which are crucial factors for *C. cassia* -producing areas in China. The main cultivated *C. cassia* is Xijiang *C. cassia*, also known as “Xijianggui,” which has the characteristics of thin skin, thick meat, rich oil, moist color, and fragrant moderately sweet and spicy taste. Guangxi *C. cassia* accounts for more than 50% of the country’s *C. cassia* planting area. Fangchenggang, Dongxing, Yulin, Guiping, and Beiliu cities are the main producing areas of *C. cassia*. Among them, the *C. cassia* cultivated in Fangchenggang and Dongxing is mainly “Fangchenggui,” whereas that cultivated in Yulin and Guiping is mainly “Xijianggui.” Therefore, based on the morphological data analysis of 71 C*. cassia* samples, we inferred that all the samples were “Xijianggui,” except for 4 samples obtained from Fangchenggang (Wei et al., 2006; Chen et al., 2014; Lin et al., 2016). Among the *C. cassia* varieties, there is also a variety from Vietnam, which is named Qinghuagui (*C. cassia* Bl forma macrophylla), famous for its large leaf shape, thick skin, and high volatile oil content. Since the morphological characteristics of the four *C. cassia* samples from Fangchenggang were similar to those of Qinghuagui, and Fangchenggang is near Vietnam, we speculated that these four samples might be from Qinghuagui. Liang et al., 2016 reported that the leaf length, leaf width, fresh leaf weight, and leaf area of the Qinghuagui family were markedly higher than those of the “Xijianggui” family by 37.03%, 26.80%, 174.88%, and 41.32%, respectively, thereby confirming our results. Genetic divergence due to genetic drift, local adaptation, and gene flow limitation may result in the phenotypic differentiation of traits (Agre et al., 2019; Arab et al., 2019).

### 4.2 Variations based on chemical components

Modern pharmacological studies have revealed that cinnamaldehyde, the main component of volatile oils, has a wide range of pharmacological effects, such as vascular dilation, anti-gastric ulcer, bacteriostasis, and anti-oxidation. Therefore, in this study, the volatile oil, moisture, and water-soluble extract contents of 71 C*. cassia* accessions were analyzed. The results showed that the volatile oil content of *C. cassia* samples from Guangxi was generally higher than those from other places except Luoding, up to 4.7%, whereas the volatile oil content of *C. cassia* samples from Luoding was the highest in Guangdong Province, up to 6.3%. The reasons for this may be related to planting history, cultivation years, or the ecological environment. Luoding, the hometown of *C. cassia* in China, has a long history of cultivation, and its ecological environment is suitable for its cultivation of *C. cassia*. The volatile oil content of *C. cassia* samples from Guangxi was generally higher than that from Guangdong Province, which may be due to the short planting period of *C. cassia* in Guangdong Province.

In a previous study by our group, we found that the planting years of Guangdong’s *C. cassia* samples were mostly 7–8 years, which might be the main reason why the volatile oil content in *C. cassia* produced in Guangdong was lower than that in Guangxi (Wei et al., 2017). Guangxi is the traditional planting base for *C. cassia* medicinal materials, and the planting years for these medicinal materials are mostly 10 years or more, mainly for clinical medicine. If the quality standards of *C. cassia* medicinal materials are to be improved, it is recommended that Guangdong’s *C. cassia* planting base increase the planting years of *C. cassia*, with 10 to 15 years deemed optimal (Xu et al., 2004). Wu et al. (2019) determined the content of cinnamaldehyde and four other volatile oil components in *C. cassia* from Guangdong and Guangxi and found that the cinnamaldehyde content in Guangxi’s *C. cassia* was generally higher than that in Guangdong’s *C. cassia*, which was also confirmed in our study (Wei et al., 2019). Furthermore, our study found that the average content of volatile oil of *C. cassia* was highest in Fangchenggang, where “Ziyougui” was also found. We speculate that perhaps because Fangchenggang is adjacent to Vietnam, some of the *C. cassia* in this area may represent the “Qinghuagui” introduced by Vietnam. Zhang. (2019) measured 45 batches of cinnamon samples from different origins using HPLC, and the results showed that the volatile oil content of cinnamon in Fangchenggang was as high as 5.2%. Qian et al. (2020) determined the cinnamaldehyde content in 11 cinnamon samples from Guangdong and Guangxi using HPLC. The results showed that the cinnamaldehyde content in Fangchenggang was 33.52 mg/g, whereas that in Luoding was only 16.23 mg/g. Wang showed that the volatile oil content of Qinghuagui (*Cinnamomum* cassia var. macrophyllum Chu) was considerably higher than that in Chinese cassia (Wang, 2011).

In a previous study, our research group found that the cinnamaldehyde content in different samples of the same batch of cinnamon medicinal materials was remarkably different and that the content difference between different thicknesses was irregular. Therefore, we studied the changes in cinnamaldehyde and volatile oil contents in the upper, middle, and lower parts of the bark of the same tree. The results showed that the contents of cinnamaldehyde and volatile oil varied greatly in the upper, middle, and lower parts of the cassia bark. The content of cinnamaldehyde in the same tree was up to two times, and the total volatile oil was up to six times, suggesting that this difference might be one of the reasons for the uneven quality of the cassia bark. In the same cinnamon tree, the cinnamaldehyde and volatile oil contents were negatively correlated with cross-sectional thickness; that is, the cinnamaldehyde and volatile oil contents increased from the near-ground part to the upper part of each cassia bark sample. The studies (Hou et al., 2013; Wang et al., 2022) have shown that the cinnamaldehyde and cinnamic acid content of *Ramulus Cinnamomi* and cinnamonic fruit is higher than that of cassia bark. *Ramulus Cinnamomi* and cinnamonic fruit are known in China as “Guizhi” and “Guiding”. “Guizhi” are branches of the cinnamon tree and “Guiding” is the tender stem of cinnamon fruit. They’re both at the top of the cinnamon tree. This may confirm our research. Therefore, it is necessary to further study the distribution of cinnamaldehyde and volatile oils in cassia bark to provide an experimental basis for the formulation of extraction technology and specification grade of cassia bark and to lay a foundation for the improvement of quality control standards.

### 4.3 Genetic diversity based on SNP

Current research indicates that domestication has less of an effect on genetic diversity reduction than breeding practices, which leads to cultivated varieties having less genetic diversity than wild counterparts (Guan et al., 2019; Wang et al., 2019; Wolfe et al., 2019). In this study, we discovered that *C. cassia* from Fangchenggang differed greatly from the others in terms of genotype, chemical composition, and morphological characteristics. This is most likely because Fangchenggang harbors ancient landraces that descended naturally from the early landraces and influenced the genetic diversification of *C. cassia* populations. However, we did not find a significant difference between the wild and cultivated types in our samples, which may be due to the fact that the wild *C. Cassia*, we were looking for were only artificially planted older trees rather than true wild *C. cassia* trees. Therefore, we need to explore wild *C. cassia* that inhabits less explored areas for further study. Wild types may be less susceptible to genetic influence from other *C. cassia* variants owing to their comparatively isolated ecological setting. The restricted genetic diversity of cultivars not only hinders the breeding process of *C. cassia*, but also elevates the likelihood of natural disasters (Luo et al., 2019; Mwale et al., 2023). Both landraces and wild-type plants may offer invaluable genetic resources for breeding initiatives, and new *C. cassia*. cultivars may be bred to diversify their genetic makeup.

Compared to other species, including rice (Peringottillam et al., 2022), wheat (Ouaja et al., 2021), corn (Rivas et al., 2022), *C. cassia* (L.), J.Presl has a substantially higher heterozygosity rate (average heterozygosity rate = 63.93%). This is most likely due to the fact that these crops have long been subjected to manual selection, which has decreased their genetic diversity. In contrast, *C. cassia* has a complex genetic basis because it has been propagated for many years using a variety of techniques, including seed, layering, and cutting propagation (Li and Jiang, 2022). The low genetic diversity within the population obtained from AMOVA could be a result of natural adaptation or extensive exchange of seeds among farmers between environments or because of the common origin of the population, which might have led to *C. Cassia* growers using the same seed continuously, without new introductions. Furthermore, we found that heterozygosity in the wild was higher than that in the cultivar types, which is consistent with findings from other species (Uba et al., 2021; Yao et al., 2012). However, the difference is not substantial. This finding may explain why *C. cassia* is propagated from one generation to another, and variation gradually accumulates, whereas wild species live in a relatively isolated setting with a lower genetic variety over extended periods of time. Therefore, they gradually become similar. Owing to the limitations of the wild individuals evaluated in this study, these are only our first conclusions about genetic variance among wild populations, and additional wild samples from various populations must be gathered for additional examination. Phylogenetic and population structure analyses demonstrated a strong positive correlation between geographical distance and gene exchange, displaying an isolated pattern within the distribution of *C. cassia*. This finding further supports the notion that there is minimal gene flow between populations, which promotes local adaptability. The clustering of *C. cassia* accessions in the current study not clearly separated the accessions based on their geographic origin, as determined by genetic distance or model simulation. The tested accessions clustered closely, both within and between neighboring locations. Using the ADMIXTURE program, all *C. cassia* accessions were assigned to three populations representing various ecogeographic regions. Cluster I was a unique and distinct Fangchenggang population, cluster II contained RG30 and RG70 from Guangdong and Guangxi, respectively, and cluster III represented populations from Deqing, Gaoyao, Luoding, Pingnan, Yulin, and Guiping. Guangdong and Guangxi were determined to have lower levels of genetic variety than Fangchenggang based on genetic differentiation in these inferred populations. The closest GD was found between Gaoyao and Deqing, while the greatest GD was found between Fangchenggang and Yulin. This is related to the distance between these species. With the exception of Fangchenggang, the GD between *C. cassia* accessions from Guangdong and Guangxi was reasonably small. In 71 samples, it was not possible to discriminate between the cultivated and wild types of the species, which is consistent with the findings of cluster analysis based on phenotypic features. According to our conjecture, the evolutionary process may have involved the ancestral population of the Guangxi area being more suited for the growth of *C. cassia*. because of the local climate and habitat, which allowed for relatively quick local breeding before spreading to Guangdong.

In summary, SNP markers provide a clear picture of the population structure and genetic diversity of *C. cassia*. This provided insightful conclusions regarding the collection, preservation, and use of Chinese *C. cassia* germplasms. The ecogeographic distribution of genetic diversity may provide information about the DNA-level spread of *C. cassia* from its genesis center to other regions of China. Our research verified that the geographic origin of *C. cassia* germplasms was linked to their population structure and that wild *C. cassia* has a more complicated genetic structure than local landraces. Furthermore, greater variation was found within the population than between populations, which directed us to gather more individuals within the same population to preserve a sufficient number of representative varieties of local *C. cassia* germplasms, especially for wild populations and landraces. In the meantime, in order to increase genetic diversity, we should gather and conserve genetic resources that come from various ecogeographic groups. From the comparatively high diversity and simple population of Fangchenggang, we were able to infer how to efficiently identify and utilize beneficial genes in local landraces and wild *C. cassia* to breed exceptional cultivars and expand their genetic base.

## Data availability statement

The datasets presented in this study can be found in online repositories. The names of the repository/repositories and accession number(s) can be found below: Bioproject accession number: PRJNA1074496.

## Author contributions

PH: Writing – original draft. JC: Writing – review & editing. ZC: Writing – review & editing. XC: Writing – review & editing. ZP: Writing – review & editing. PD: Writing – review & editing.
