# Supplementary material for: Exploring genetic diversity and population structure in Cinnamomum cassia (L.) J.Presl germplasm in China through phenotypic, chemical component, and molecular marker analyses
Source: Front Plant Sci. 2024 Jul 3;15:1374648. doi: 10.3389/fpls.2024.1374648 (PMC11270630; doi:10.3389/fpls.2024.1374648)
Supplement: Supplementary file 1 [file DataSheet_1.docx]

Supplementary Material

**Exploring genetic diversity and population structure** **in *Cinnamomum cassia* (L.) J.Presl germplasm in China through phenotypic, chemical component, and molecular marker analyses**

**Panpan Han^1^, Jinfang Chen^1^, Zeyu Chen^1^, Xiaoying Che^1^, Ziqiu Peng^1^, Ping Ding^1*^**

*** Correspondence:** Ping Ding: [dingping@gzucm.edu.cn](mailto:dingping@gzucm.edu.cn)

# Supplementary Figures

1. **(B) (C)**

**Supplementary Figure 1.** The moisture, water-soluble extract and volatile oil content of *C. cassia*. **(A)** The content of volatile oil in 71 *C. cassia* samples. **(B)** The content of moisture in 71 *C. cassia* samples. **(C)** The content of water-soluble extract in 71 *C. cassia* samples.

**Supplementary Figure 2.** Changes of cinnamyl alcohol and 2-methoxycinnamaldehyde contents in cinnamon bark of different thicknesses.

**Supplementary Figure 3.** The distribution of the minor allele frequency (MAF).

**Supplementary Figure 4.** The distribution of polymorphism information content (PIC).


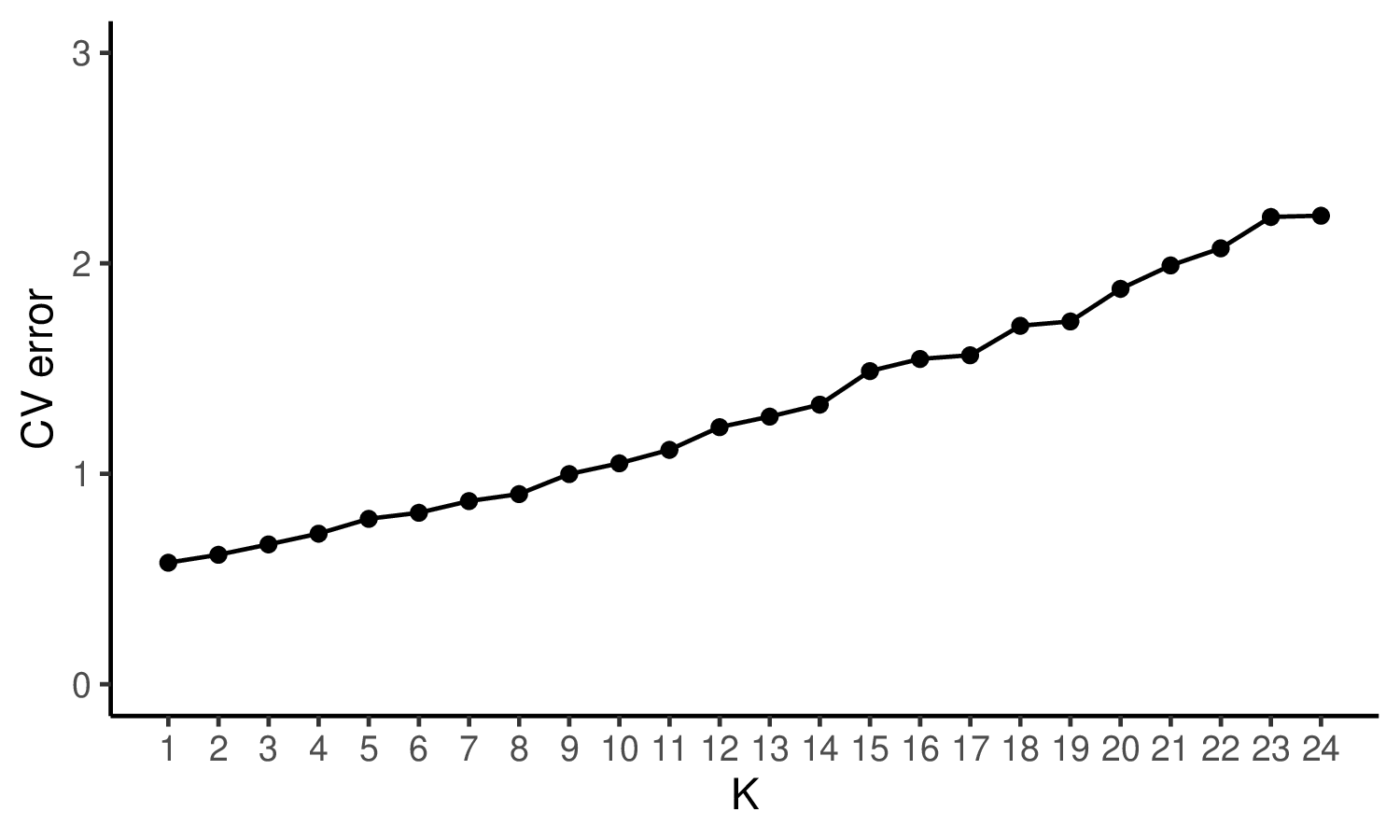


**Supplementary Figure 5.** A broken line diagram of the K value and CV error.
